# Radiofrequency Ablation and Concomitant Sclerotherapy for the Treatment of Varicose Veins (VV): Perspectives from a Developing Country

**DOI:** 10.3400/avd.oa.21-00027

**Published:** 2021-12-25

**Authors:** Muhammad Yousuf Memon, Ilyas Sadiq, Safdar Ali Malik, Muhammad Bin Zulifqar, Muhammad Saad Malik, Muhammad Hammad Malik

**Affiliations:** 1 Section of Interventional Radiology, Division of Shaheed Muhtarma Benazeer Bhutto Trauma Center, Civil Hospital, Karachi, Pakistan; 2 Division of Vascular and Endovascular Surgery, Allama Iqbal Medical College, Jinnah Hospital, Lahore, Pakistan; 3 Alnoor Diagnostic Center and Institute of Radiology, Lahore, Pakistan; 4 CMH Lahore Medical College, Combined Military Hospital, Lahore, Pakistan

**Keywords:** venous insufficiency, ablation, sclerotherapy, developing country, endovenous heat-induced thrombosis

## Abstract

**Objectives/Background**: With decreased patient downtime and reduction in health expenditures, endovascular treatments have become popular for the treatment of venous insufficiency. In this study, we assessed the outcomes of using radiofrequency ablation (RFA) and sclerotherapy for refluxing veins and incompetent perforators in a developing country.

**Materials and Methods**: Subjects were selected from an ongoing registry from October 15, 2015 to April 5, 2018. Patients were followed up until 6 months. Pre- and post-procedural Clinical, Etiologic, Anatomic, and Pathophysiologic (CEAP) scores were compared, and complications were documented and treated accordingly.

**Results:** In total, 102 limbs (n=97) with 76 great saphenous veins (GSVs) and 26 small saphenous veins (SSVs) underwent RFA, with 79% undergoing concomitant sclerotherapy. Mean follow-up time was 188 days (±33.16). Moreover, 59% were males and 41% females. At the end of follow-up, 99% of the legs had complete occlusion. Pre- and post-procedural CEAP scores were 4.21±1.5 and 3.36±1.7, respectively (p-value <0.001). Endovenous heat-induced thrombosis (EHIT) types 1, 2, 3, and 4 were found in 8.8%, 3.9%, 1.9%, and 0% of the legs, respectively. Most common complications were pain and tenderness (51%), bruising (18%), and paresthesia (7%).

**Conclusion**: RFA and sclerotherapy have proved to be safe and efficacious. Computed tomography (CT) venous mapping aids in delineating complex venous anatomy and in ruling out deep vein thrombosis (DVT) in cases with discrepancy on Doppler ultrasound. Strict compliance of procedural and post-procedural protocol can assure promising results and futuristic value.

## Introduction

With the advent of endothermal ablation, strategies in treating venous pathologies have significantly revolutionized. Robust evaluation has introduced more sophisticated options like ablation therapy and sclerotherapy and allowed for the transition of care into a more ambulatory setting. As it demonstrates lesser patient downtime and reduced health expenditures, they have become the more popular sought out option compared to conventional surgery since the early 2000s.^1–3)^

Chronic venous insufficiency can have a significant impact on the population, both quantitatively and qualitatively.^4)^ In Pakistan, the prevalence of chronic venous disease is at an average figure of 34.8%, with predominantly higher distribution in men (36.4%) than in women (33.0%).^5)^

Rates of reflux-associated complications such as recurrent varicosities and ulcers vary from 20% to 60%.^6,7)^ Research suggests that persistent reflux in superficial veins of the lower limb acts as a sequela to the incompetency of perforator veins which, in concert, lead to a poorer prognosis of the disease.^8,9)^

In this study, we aimed to assess the outcomes of using radiofrequency ablation (RFA) with concomitant ultrasound-guided sclerotherapy (USGS) in the management of refluxing lower limb veins and incompetent perforators.

## Materials and Methods

### Definitions

Due to overlap between previous classifications such as that of Lawrence and Kabnick et al., there has been heterogeneity in reporting and outcomes of endodermal thrombus extension. Henceforth, we used the American Venous Forum (AVF) endothermal heat-induced thrombosis (EHIT) classification^10)^ in terms of defining the procedure-induced thrombosis. We defined recanalization as 5 cm or greater segment of flow in a previously treated vessel. Severity of varicose veins (VV) was ranked using the Clinical, Etiologic, Anatomic, and Pathophysiologic (CEAP) classification system.^11)^

### Data collection and inclusion criteria

Subjects were added prospectively in an ongoing registry from October 15, 2015 to April 5, 2018. RFA was performed using ClosureFAST catheter (©2012 Covidien, Mansfield, MA, USA) on the great saphenous veins (GSVs) and small saphenous veins (SSVs). The 12-mm size limit (applied on first-generation devices as submitted by U.S. Food and Drug Administration) was not used in our study with the new ClosureFAST catheter, as studies have shown no effect on closure rates.^12)^ Patients’ demographic and clinical data included age, gender, height, weight, body mass index, presenting symptoms, and history of deep vein thrombosis (DVT). Three-dimensional volume-rendered images were obtained using computed tomography (CT) venography in patients with high suspicion of DVT. Individuals with a reflux of 0.5 s or greater were selected. Individuals with a previous or current diagnosis of DVT, extremely tortuous (GSV), CEAP score of 0–1, and women known to be pregnant were excluded from analysis.

A venous reflux was elicited on color Doppler (Voluson E8 (GE Healthcare Austria GmbH & Co OG, Tiefenbach, Austria)) in the saphenofemoral, saphenopopliteal, or truncal vein while patients carried out the Valsalva maneuver in standing or in a 15° reverse Trendelenburg position. After attaining a near bloodless field, soft tissue planes between the saphenous fascia and muscularis fascia were infiltrated with a homogenous layer of intumescent solution (500 ml=40 ml xylocaine +10 ml of 2% sodium bicarbonate +450 ml of normal saline) along the length of the vein being ablated.

**Figure 1:**
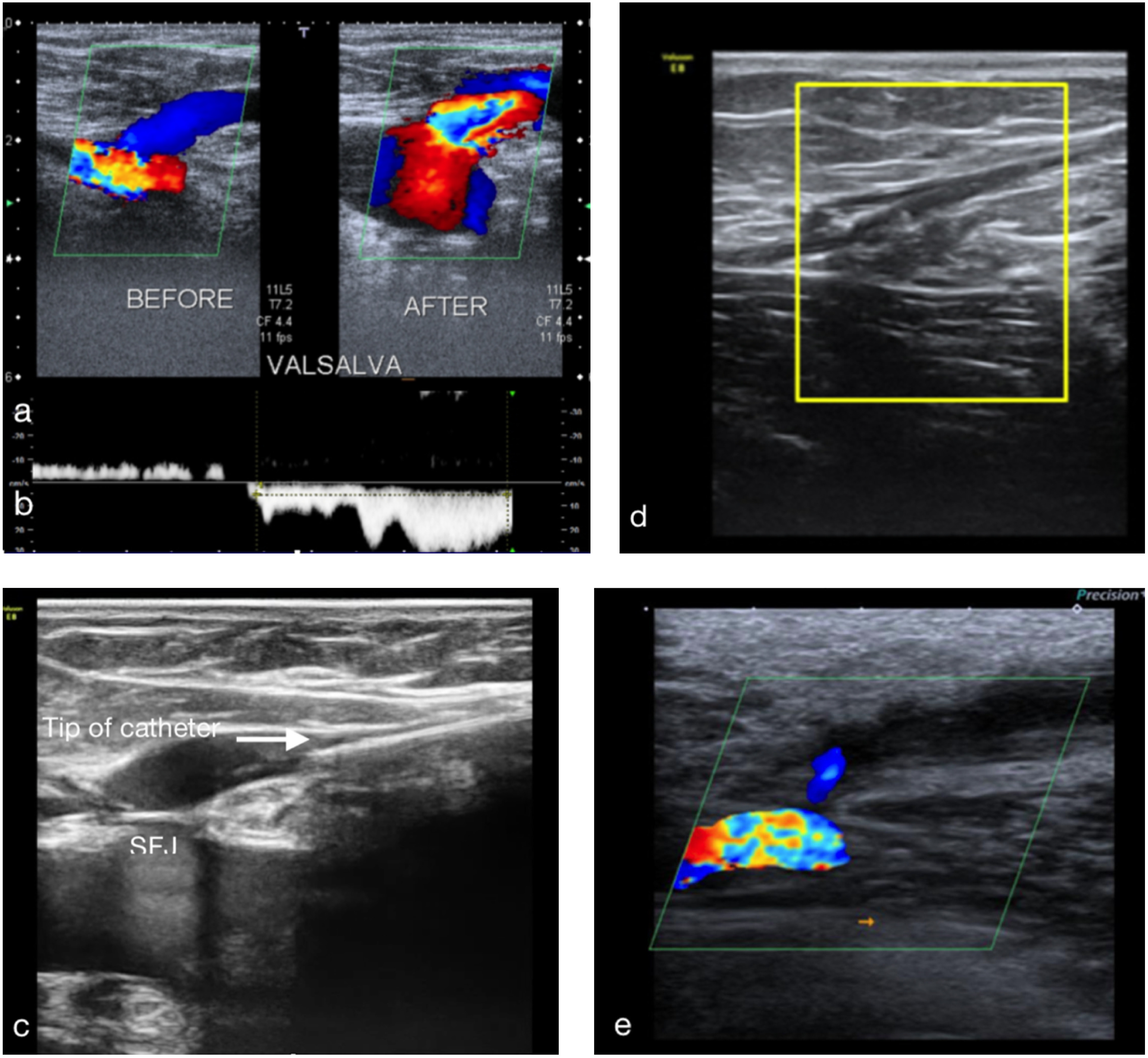
Fig. 1 Radiofrequency ablation (RFA). (**a**)–(**c**) Case 1-A 46-year-old male with refluxing right great saphenous vein (GSV). Pathologic reflux after the augmentation maneuver with Doppler image showing change in color, increase in caliber of GSV (**a**); reflux time >0.5 s observed on Doppler (**b**); tip of RFA catheter seen approaching the saphenofemoral junction (SFJ) (>2 cm away from SFJ) (**c**); (**d**)–(**e**) Case 1 continued. Immediate post-RFA ultrasound shows thrombosed GSV (**d**); Repeat Doppler at 24 h confirms the absence of blood flow in the GSV (**e**).

Using Seldinger technique, an ultrasound-guided wire was inserted around the knee joint or from the lower calf up to 2 cm short of saphenofemoral junction (SFJ) or saphenopopliteal junction, respectively. A 6 F vascular sheath was progressed over the guide wire followed by catheter insertion. The operated vein was ablated in 5–6 cm segments for 20 s each at a temperature of 120° using a ClosureFAST catheter until the distal part was visualized to be in direct contact with the skin with absent blood flow on Doppler (**Fig. 1**).

**Figure 2:**
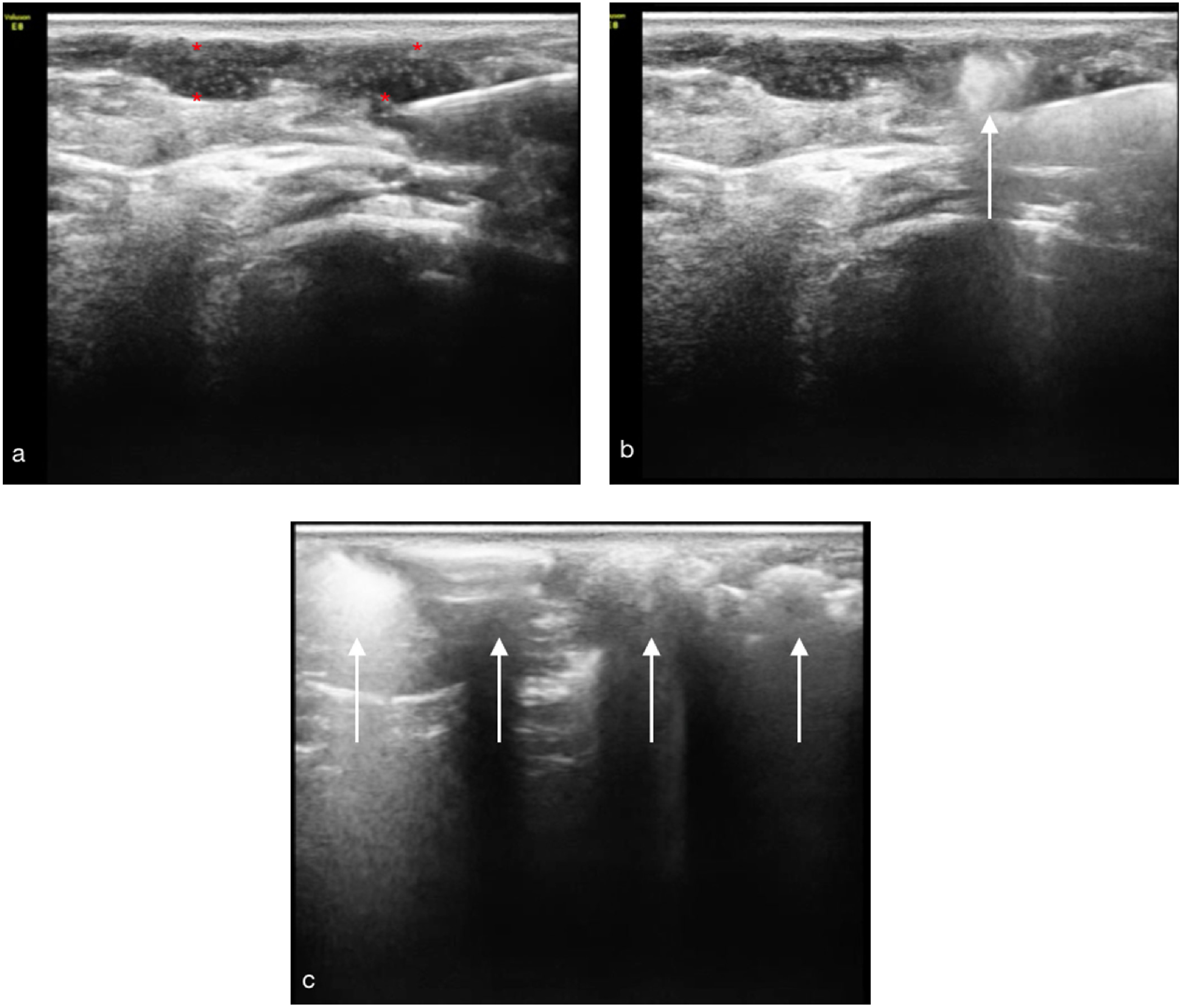
Fig. 2 Ultrasound-guided injection sclerotherapy (STD). (**a**)–(**c**) Case 2-A 32-year-old male with an insufficient leg perforator with associated local skin discoloration and ulceration. Tip of the catheter seen in dilated perforator (diameter >3 mm) at the level of inferior thigh (**a**); Color Doppler ultrasound (US) shows the tip of the catheter with a bright echo of STD (**b**); US shows visible obliteration of the perforator lumen and multiple segments of hyperechogenicity (arrows) throughout the perforator at the end of procedure (**c**).

RFA was performed concomitantly with USGS in patients who had co-existing perforator incompetence (**Fig. 2**). With the help of color flow Doppler scanning, we identified perforator veins with diameter ≥2 mm and the presence of outward flow or bidirectional flow. Elicitation of reflux was done by “manual compression and rapid release” technique. Incompetence was defined using a cutoff value of >0.5 s of refluxing time on spectral analysis in truncal veins with >2 s being considered severe incompetence. All significant perforator veins observed on Doppler scanning were marked before the procedure. The most superficial region of the perforator vein was chosen for injection of sclerosing agent to avoid injecting into the adjacent artery. The procedure was aborted in case of extravasation of sclerosing agent or if high resistance was encountered. Injection sclerotherapy was done with sodium tetradecyl sulfate (Setrol (Samarth Pharma Pvt. Ltd., Mumbai, India)). The foam was produced using Tessari method, by mixing Setrol with air in a ratio of 1 : 4. A second duplex scan at the end of the procedure confirmed vein closure and vein fullness secondary to RFA and USGS, respectively.

### Post-procedural management

Compliance with post-procedural management protocol was universal. Operated limbs were wrapped in bandages, and patients were prescribed prophylactic Nonsteroidal anti-inflammatory drugs (NSAIDs) and antibiotics. Bandages were substituted with class II compression stockings 48–72 h later. Follow-ups were scheduled at 24–72 h, 1 week, 1 month, 3 months, and 6 months. Patient satisfaction was gauged by a simple question and answer criteria similar to the one used by Merchant et al.^13)^

### Statistical analysis

All data were recorded in hardcopy format and converted for analysis using the Statistical Package for Social Sciences (SPSS, version 21). Paired sample t-test was used to compare pre-procedural and post-procedural CEAP scores. A p-value of <0.05 was considered statistically significant (with a 95%CI).

## Results

Patient particulars and CEAP classification are summarized in **Table 1**. Between October 15, 2015 and April 5, 2018, a total of 102 RFA procedures were performed on 76 GSVs and 26 SSVs of 97 patients. Concomitant perforator sclerotherapy was performed in 79% (n=81) of the legs. Mean age was 54 years ±13.8 (27–88 years old) and included 59% (n=57) males and 41% (n=40) females. The most common indication for the procedure was leg ulcers (n=43), with 86% (n=37) of them being actively bleeding and discharging. Pre-procedural and post-procedural (1 year) CEAP scores were 4.21±1.5 and 3.36±1.7, respectively, with a difference of 0.84±0.59 (p<0.001).

**Table 1:** Table 1 Patient demographic details (n=102)

Characteristics	n±SD
No. of patients	97
Age (years)	54.5±13.8 (27–88)
Men	57
Women	40
No. of legs	102
Bilateral veins	5
Concomitant sclerotherapy (legs)	81
Vein distribution	
Great saphenous vein (GSV)	76
GSV diameter (mm)	9.8±3.0
GSV length (cm)	32.6±6.1
Short saphenous vein (SSV)	26
SSV diameter (mm)	5.7±1.6
SSV length (cm)	20±5.4
CEAP* classification	
C2–C3	40
C4–C6	62
CEAP scores	
Pre-procedural	4.19±1.58
Post-procedural**	3.36±1.7
Difference	0.84±0.59
P-value	<0.001

*CEAP classification: Clinical, Etiologic, Anatomic, and Pathophysiologic classification. **6 months of follow-up (188 days ±33.1).

### Closure patterns and EHIT

In total, 99% (n=101) of the legs had complete occlusion of the GSV confirmed by duplex ultrasound on their first post-procedural visit. Duplex ultrasonography demonstrated complete vein occlusion in 100% of SSVs (n=26) and 97% for GSVs (n=75) at 6 months (mean time 188 days ±33.1). EHIT types 1, 2, and 3 were seen in 10.7% (n=11), 2.9% (n=3), and 0.9% (n=1) of the legs, respectively. No cases of EHIT type 4 closure pattern that completely occluded the femoral vein were observed. Those who had EHIT types 2 and 3, which required anticoagulation, had a mean age of 32.75±3.30 years compared with a mean age of 55.5±13.6 years for those with a lower level of closure. Two cases of recanalization were documented in refluxing GSVs with flow segments of 20–22 cm and 15–17 cm from SFJ to occlusion stump at day 2 and 13 months, respectively. Reflux was re-elicited at the saphenofemoral junction in both cases. Both GSVs had been treated with RFA and USGS.

### Post-procedural complications

A list of complications recorded at follow-ups is shown in **Table 2**. No cases of pulmonary embolism or venous thromboembolism (VTE) were documented according to our 6-month follow-up. In total, 51% (n=52) patients complained of pain and tenderness at 24 h, followed by bruising at the site of catheter insertion (17.6%). One patient reported persistent pain at 30-day follow-up. Pain was in the posterior mid-thigh region of their operated limb. Moreover, 6.8% (n=7) of patients reported paresthesia, while 2.9% (n=3) reported thrombophlebitis. No sign of infection or burns was seen. One patient returned in the first month with persistent symptoms of venous insufficiency (recurrent swelling, lipodermatosclerosis, and persistent ulceration).

**Table 2:** Table 2 Post-procedural complications (n=102)

Complications, n (%)^1^	
EHIT^2^	
Type 1a	2 (1.9%)
Type 1b	9 (8.8%)
Type 2	3 (2.9%)
Type 3	1 (0.9%)
Type 4	—
Single episode of venous thromboembolism^3^	—
Recanalization	2 (1.9%)^4^
Thrombophlebitis	3 (2.9%)
Infection	—
Bruising	18 (17.6%)
Pain and tenderness	52 (50.9%)
Paresthesia	7 (6.8%)

^1^ Four patients were lost to follow-up at 6 months. ^2^ American Venous Forum (AVF) EHIT classification. ^3^ Deep vein thrombosis, pulmonary embolism, etc. ^4^ Day 2 and 13 months.

## Discussion

Globally, multiple reports have recommended endovenous procedures due to lesser patient downtime, quicker return to normal activity, and less post-procedural complications as opposed to conventional surgery.^14,15)^ A comparison by Rasmussen et al. revealed that the number of legs that exhibited recurrent VVs at 1-year follow-up in the surgical stripping, ultrasound guided foam sclerotherapy, endovenous laser treatment (EVLT), and RFA group were 16 (14.8%), 17 (13.8%), 14 (11.6%), and 9 (7.3%) of the legs, respectively.^16)^ Among endovenous techniques, we chose RFA over EVLT as the RECOVERY Study supported the claims of statistically lower rates of complications in RFA than in EVLT.^17)^

**Figure 3:**
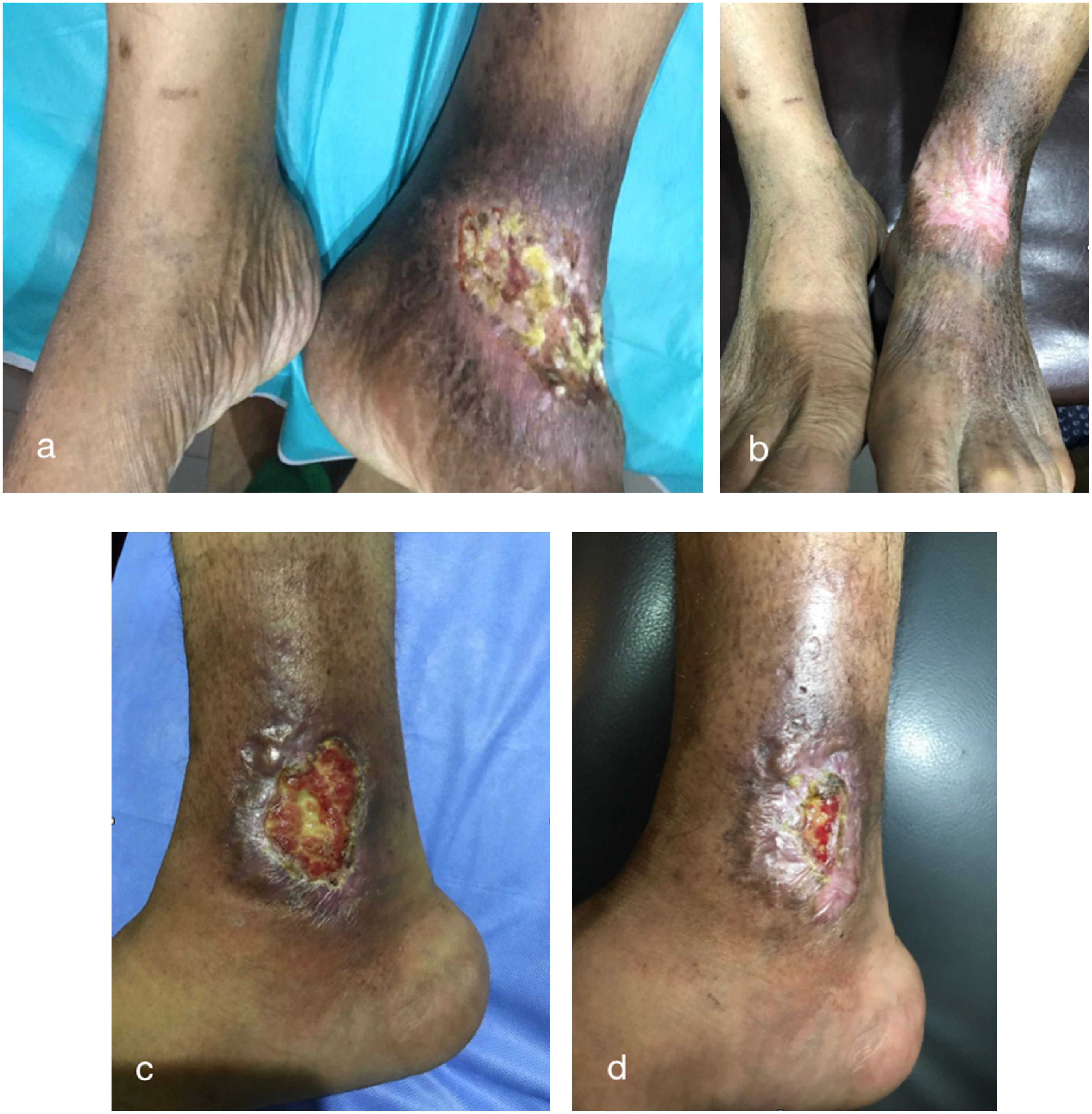
Fig. 3 Before and after results comparison. (**a**)–(**b**) Case 3-A 53-year-old male with refluxing left great saphenous vein (GSV) and incompetent perforators below the left knee. Before: An actively bleeding venous ulcer (CEAP 6) with purulent discharge on the anteromedial surface of the left ankle with extension onto the dorsal surface of the left foot (**a**); after: following radiofrequency ablation (RFA) and injection sclerotherapy (USGS), an 8-week follow-up shows a completely healed ulcer (CEAP 5) with significantly reduced inflammation and residual skin discoloration (**b**); (**c**)–(**d**) Case 4-A 44-year-old male with refluxing right GSV and incompetent perforators below the right knee. Before: An actively bleeding venous ulcer (CEAP 6) with purulent discharge on the medial malleolar surface of the right limb (**c**); after: following RFA and injection sclerotherapy (USGS), a 3-week follow-up shows a near-complete (>80%) healed ulcer (CEAP 5) with significantly reduced inflammation (**d**).

The primary success rate depended upon post-procedural clinical resolution of truncal varices with absent flow on duplex evaluation and no remaining reflux. We observed an intraoperative failure rate of 0% with no detectable segments of flow on duplex scanning during treatment which is comparable to other studies.^1)^ At the end of 6 months, 99% (n=101) showed successful venous occlusion with visibly appealing results when compared visually (**Fig. 3**). The success rates in this present study compares well with the rates published in most other studies.^18,19)^ Calcagno et al.^12)^ reported a similar closure rate of 98% in GSVs less than or equal to 12 mm and 100% in GSVs greater than 12 mm. Moreover, data collected at Zagazig University, Egypt, concluded that truncal sclerotherapy below the knee and RFA above the knee of GSVs showed lesser complications and more effective closure rates at 12 months than monotherapy with RFA alone.^20)^

Recanalization was seen in two patients (day 2 and day 394). The first cause may have been due to non-adherence to post-procedural protocol. The patient stated not switching to compression stockings after 48 h, which may have resulted in failure of venous closure. The patient underwent the redo procedure and had two additional sclerotherapy sessions for perforator occlusion in the month following the redo procedure. According to the last follow-up (108 days), the patient had documented the evidence of complete obliteration of GSV (>2.5 cm from SFJ) with the sonographic evidence of absent flow in operated perforators. According to our 6-month follow-ups, no other case of recanalization was documented in the treated veins. A similar study by Weiss and Weiss^21)^ reported 98% (137/140) of patients with successful vein occlusion at 6 weeks and no new cases of recanalization in the 1-year follow-up. The second cause of recanalization may have been due to excessive weight gain as the patient had gained an additional 40 kg since their last visit (166 days post-op). The patient was offered the redo procedure, but they were lost to follow-up.

Some studies have reported lower occlusion rates after RFA. Merchant et al.^13)^ in his study of 286 patients from 30 clinical sites reported an occlusion rate of 83.6% at 12 months and 85.2% at 24 months, respectively. It is possible that a lower ablation temperature and the type of catheter used might be the cause of variance in occlusion rates. Merchant’s study used a ClosurePlus catheter with a temperature of 85°C±2°C, while we used ClosureFast at an ablation temperature of 120°C. Choi et al.^22)^ drew out a similar conclusion when comparing the two types of catheters. Furthermore, a more circumferential collagen contraction is observed in ClosureFast catheter due to even transmission of temperature as compared to ClosurePlus, attributing to lower rates of recanalization.

Earlier reports observed that endothermal ablation led to an increased risk of DVTs observed post-procedurally (0% to 8%).^23,24)^ However, later publications redefined these thrombi as thrombus extension (EHIT) rather than DVT as they were recognized as separate entities.^25,26)^ Although findings of previous studies suggest that EHIT-1 does not seem to be a relevant clinical finding, we noticed it was more pronounced in our study (8.8%) when the first two cycles of ablation were done at the same location from the SFJ rather than 1 cm apart. It is plausible to assume that the same principle played a role in EHIT of higher grades. Immediately withdrawing the catheter 2 cm distally after a single cycle of ablation, as opposed to two, significantly changed outcomes as no new cases of EHIT were observed thereafter, which has been highlighted in previous studies.^27)^ Additional techniques such as extreme Trendelenburg position as well as abundant tumescence may prove helpful.

Legs with EHIT type 2 (2.9%) were managed via therapeutic anticoagulation (low-molecular-weight heparin) and weekly surveillance.^10)^ Mean regression time was 8.6 days ±1.5.^3)^ Patients were instructed to continue wearing compression stockings. Similar results were found by Lawrence et al.^28)^ with 2.6% (13/500) developing DVTs post-procedurally with patients recovering completely within 1–2 months. Our patients were not given anticoagulation pre-procedurally, to avoid any interference with thrombotic vessel occlusion. Another factor attributable to the low risk of DVTs in our study may be the prophylactic use of aspirin 75 mg (Loprin) with its anti-platelet effect. EHIT type 3 (0.9%) resolved within 10 days of standard anticoagulation. The patient was kept under weekly surveillance until thrombus retraction to the SFJ. It is difficult to preclude any meaningful conclusions toward identifying predisposing risk factors involved in EHIT owing to the very low number of events and the lack thereof of substantial correlation and statistical power.

In total, 30 patients came for an additional session of sclerotherapy in the month following RFA for residual perforators and accessory venous closure. All patients had complete closure of GSV from previous RFA session. This positively reinforced ulcer healing and accounted for more desirable cosmetic outcomes.^16)^

One patient (29-year-old male) returned in the first month with recurrent swelling, lipodermatosclerosis, and persistent ulceration. Medical records were negative for any previous hospital admissions, and initial Doppler studies before conducting RFA did not reveal any signs of DVT from the femoral vein and downward. Investigative CT venography showed scarring and residual narrowing of segments of the external iliac vein, and a diagnosis of post-thrombotic syndrome was made. These changes may have been due to a previous diagnosis of DVT that was not declared by the patient nor detected on Doppler imaging. Luminal narrowing, central thread-like lesions, loss of augmentation of flow, and loss of respiration phasicity should prompt suspicion of an occult DVT higher up in the venous vasculature and should be investigated via CT venogram if Doppler proves to be futile. Subsequently, as an additional preliminary step, we added CT venography in all such suspicious cases despite negative medical records. Use of CT venography has been shown to have higher diagnostic accuracy.^29)^ It allowed better visualization and delineation of venous vasculature in close proximity to the pelvic, abdominal veins, and inferior vena cava. Patients with current or prior DVT were referred to interventional radiologists and not followed up later.

With the instillation of tumescent anesthesia, complications such as iatrogenic nerve injury have virtually vanished.^13,21)^ In our study, only one (0.9%) patient complained of pain and paresthesia that persisted for 3 months. A neurological examination at 3 months revealed the cause of pain to be a degenerative process of disc prolapse and not the RFA procedure itself. Thrombophlebitis occurred in 2.9% (n=3) of our patients and was addressed using symptomatic measures. Patients were prescribed NSAIDs and encouraged to employ hot fomentation and compression hosiery after the procedure leading to near-complete resolution at 1-week follow-up, comparable to other studies.^1)^ With the help of NSAIDs and analgesics, pain was reduced in almost 70% (n=24) of patients in the first week, with numbers reaching 90% (n=34) in the following month.

To the best of the authors’ knowledge, this is the first reported and first large-scale study of RFA and USGS of VVs at a single center in Pakistan. One shortcoming of this present study is that, for practical reasons, the treatment and follow-up examinations were not blinded. Only CEAP classification was used for grading of patients, whereas various studies have also used Venous Clinical Severity Score (VCSS).^16)^ For quality of life, more objective-based tools like the Aberdeen Varicose Vein Symptom Severity Score, Medical Outcomes Study Short Form 36 health-related QoL score (SF-36) and visual analog scale can be used. Data comprised of the RFA procedure only; hence, direct comparison could not be drawn with other treatment modalities.

### Recommendations

Moreover, while we have provided outcomes up till 6 months after treatment, we encourage further studies which evaluate even longer outcomes at 1-year and 2-year follow-ups. Comparison of cost variability between RFA and conventional surgical options could not be done but is encouraged for the future.

## Conclusion

Application of RFA for ablating refluxing venous segments and the concomitant use of sclerotherapy foam for the closure of perforator veins have been determined to be an effective approach for the treatment of VVs with promising outcomes at 6 months of follow-up in countries with lower-middle income settings. We have learned that if conventional Doppler proves insufficient in fully delineating complex and/or aberrant venous anatomy, CT venous mapping could aid in acquiring better visualization. Furthermore, specific areas such as iliac DVT and DVT in the profunda femoral vein, which are not usually imaged with ultrasound, can readily be investigated with the use of CT venography. We hope that our findings help physicians and encourage the use of minimally invasive techniques such as RFA for the management of chronic venous insufficiency in the developing world.
