# Supplementary material for: Radiofrequency Ablation and Concomitant Sclerotherapy for the Treatment of Varicose Veins (VV): Perspectives from a Developing Country
Source: Ann Vasc Dis. 2021 Dec 25;14(4):341–7. doi: 10.3400/avd.oa.21-00027 (PMC8752919; doi:10.3400/avd.oa.21-00027)
Supplement: Supplementary Data [file avd-14-4-oa.21-00027-s001.pdf]

Class Definition
I Thrombus without propagation into the deep vein
a. Peripheral to superficial epigastric vein
b. Central to superficial epigastric vein, up to and including the deep vein junction
II Thrombus propagation into the adjacent deep vein but comprising <50% of the deep vein lumen
III Thrombus propagation into the adjacent deep vein but comprising >50% of the deep vein lumen
IV Occlusive deep vein thrombus contiguous with the treated superficial vein

Supplementary Table 1. American Venous Forum (AVF) endothermal heat-induced thrombosis (EHIT) classification

Kabnick LS, Sadek M, Bjarnason H, et al. Classification and treatment of endothermal heat-induced thrombosis: Recommendations from the American Venous Forum and the Society for Vascular Surgery. *J Vasc Surg Venous Lymphat Disord*. 2021;9(1):6-22. doi:10.1016/j.jvsv.2020.06.008
